# P130: Assessment of hand hygiene practices at the two children's hospitals in Greece

**DOI:** 10.1186/2047-2994-2-S1-P130

**Published:** 2013-06-20

**Authors:** S Kouni, K Mougkou, G Kurlaba, C Nteli, A Lourida, S Maroudi-Manta, T Zaoutis, S Coffin

**Affiliations:** 1 The Stavros Niarchos Foundation-Collaborative Center for Clinical Epidemiology and Outcomes Research (CLEO), University School of Medicine, Athens, Greece; 2 Pediatric Intensive Care Unit, Aglaia Kyriakou Children's Hospital, Athens, Greece; 3 Division of Infectious Diseases, Department of Pediatrics, UPENN School of Medicine, Philadelphia, PA, USA

## Introduction

Hand hygiene (HH) is one of the basic components of the infection control program and is frequently considered synonymous with hand washing. The use of a waterless, alcohol-based hand rub (ABHR) is more effective, saves time and promote better compliance than hand washing.

## Objectives

The aim of the study was to estimate the current HH practices in order to schedule the future interventions.

## Methods

Observational HH data were collected from 13 wards in 2 pediatric hospitals in Athens, including medical/surgical, oncology/transplant (BMTU), and intensive care units (ICUs), during 65, 1-hour observations periods, from October 2012 to January 2013. HH opportunities and attempts were designated as appropriate or inappropriate per WHO criteria.

## Results

A total of 1271 HH opportunities were identified during the observation period. Overall HH compliance was 33% (417/1271) of which 58.8% were appropriate. Compliance differed by role: nurses (49%), physicians (24%) and others (19 %) (p≤0.001). Healthcare workers (HCWs) and visitors were more likely to use soap and water (76.1%) compared to ABHR (23.9%) and no significant difference was detected among these groups (p=0.330). In regards to type of department, the use of ABHR was found to be strongly higher in Surgical wards (71.8%) compared to the rest of wards which this rate ranges from 11.1% in NICUs to 33.3% in emergency departments (p<0.001). The HH procedure was appropriate in 63.4% and 45.4% among those used soap and water and ABHR, respectively (p=0.002). The most commonly identified HH opportunities were after child contact (381), before child contact (376), after contact with child’s surroundings (358) and before aseptic procedure (95). Despite the fact that all HCWs use more often hand washing, a minor number of HH opportunities (61) were identified after contact with body fluids, the step of HH which demands this HH method.

## Conclusion

A low level of HH compliance and use of ABHR was observed. The education of the appropriate use of ABHR must be the main intervention for hand hygiene in these health care facilities.

## Disclosure of interest

None declared

